# Supplementary material for: Communicating the risk of contracting Zika virus to low income underserved pregnant Latinas: A clinic-based study
Source: PLoS One. 2020 Nov 20;15(11):e0241675. doi: 10.1371/journal.pone.0241675 (PMC7679023; doi:10.1371/journal.pone.0241675)
Supplement: Attachment — Submitted filename: Response to Reviewers.pdf [file pone.0241675.s001.pdf]

Response to Reviewers:
Communicating the Risk of Contracting Zika Virus to Low Income Underserved Pregnant Latinas: A Clinic-based Study

We received reviews from only one individual. The following are our responses (blue text) to reviewer's Questions. We used track changes option with pink color to mark edits and inserts in the text.

Reviewer #1:
The paper entitled "Communicating the Risk of Contracting Zika Virus to Low Income Underserved Pregnant Latinas: A Clinic-based Study" addresses as important and often overlooked topic in Zika research, communication of risk to higher risk populations. Overall this paper is nicely written and provides insights into the clinician-patient interactions during and following the Zika pandemic. There are several things that could be done, however, that would improve the manuscript.

Introduction:
- It Is important to contextualize the research presented in the introduction with the dates and time-frames that they address. Knowledge and communication transformed throughout the pandemic and many of the articles cited appear to be from early on in the pandemic.
- Authors could update their introduction with findings from a broader geographic context; there are a number of other studies on Zika knowledge, attitudes and practices that could provide some useful background that were focused in Florida.

RESPONSE:
Added pertinent text as requested. We have included recent information concerning Zika knowledge, attitudes and practices under introduction, see pages 5 and 6.

Methods:
- More details on study site are needed – north Texas county, distance from border, additional characteristics about the population in the country would be helpful; percent below poverty, percent born in other countries.

RESPONSE:
Added pertinent text as requested concerning the county and the state under participants section, see pages 7 and 8.

- More description of the recruitment strategy and how they determined this clinic served 85% of Latina women in the county are warranted. How did they ensure women did not take the survey multiple times?

RESPONSE:
Added pertinent text as under study participants, see page 7.

- October 2016- Feb. 2018 is a long time period. It is important to include a variable that divides the data into time periods (no more than three given the smaller sample size) to examine how these data changed

over time. My guess is Zika information dissemination feel rapidly throughout 2017. This time frame was very late in the pandemic and the timing should be discussed.

RESPONSE:

The study began in October 2016; however, the actual data collection was done between January 2017 and February2018. The first three months of the study were spent in locating the clinics and obtaining their approval, getting the study approvals from the university Institutional Review Board, and recruiting the mothers. We have included the text to clarify this timeline. See page 8.

- Justify the sample size involved in this study and any information pertaining to this - e.g. was a power calculation performed prior to participant recruitment? Also, please refer to any post-hoc corrections following multiple comparisons that were performed or explain why these were not included.

RESPONSE:

The sample size was determined based on the funding available. However, we have performed post-hoc power analysis and arrived study power at 0.92, which is high. We also considered significance level of adjusted alpha .01 (see page 9) for multiple comparisons. Adjusted odds ratios (AOR) were calculated, and level of significance for testing of each model was set to an alpha of .05, and 95% confidence intervals (CIs) are reported. However, we set a higher significance threshold (.05/5 independent predictors in the model= adjusted alpha = .01) for individual comparisons to compensate for the number of inferences being made, see page 10.

- While the single variable approach is great to determine the domains of knowledge that are weaker, an aggregate score could reveal patterns in overall general knowledge that could be further useful. Suggest conducting and including in supplementary data.

RESPONSE:
We have created an aggregate score by summing the knowledge items in our earlier analyses. Since there was not much variation, (only less than 5% (n=14) reported having knowledge on two items or less. Hence, we did not report. As per the reviewer's comment, we have now included the results now in the revised version. See page 12.

- The explanatory variables are quite limited and may be subject to confounding.

RESPONSE:
Explanatory variables include individual and demographic factors that are typically collected in any survey (age, education, marital status, income, employment, and pregnancy-intentional/unintentional). We are not

sure what other demographics the reviewer was referring to. It would have been nicer if he or she had mentioned them. We have used all the variables collected in the study.

- Table 2 – items g and j should still be presented.

RESPONSE:
We have now included results from the LR for items g and j in Table 2. See page 16.

Discussion
- The low rates of travel may explain why the women were not feeling at risk
- Single moms are only significantly lower on the planning and caring to postpone pregnancy – this is likely confounded by unintended pregnancy, if they were not planning to become pregnant then they would not be likely to postpone.

RESPONSE:
We have now included pertinent information in the revised manuscript (page 21).

- Why did they not look at source of knowledge and knowledge associations, this would be more straightforward and address some of the questions about how women receive knowledge and their misperceptions

RESPONSE:
The response categories for the question that inquired sources is "check all that apply", meaning the responses are not mutually exclusive. A person can obtain information via multiple sources simultaneously. For this reason, we cannot test associations between source of knowledge and knowledge.

LIMITATIONS
The study predicates that there is a gap in knowledge in border communities yet the limited data on the area suggests this is not a typical border community where greater than 50% of the population are typically LatinX. Some context and discussion about the site will allow readers to understand the generalizability to the border communities.

RESPONSE:
Added pertinent text as requested concerning the generalizability under limitations. See pages 23-24.

- Limitations of the dependent variable with yes/ no responses should be discussed including potential response bias. Individuals may have a tendency to over report their prior knowledge.

RESPONSE:
We have now included this limitation in the text in page 24.

- Despite pilot testing the last knowledge question about delaying pregnancy is confusing. While interesting, it may be hard to interpret given it is a three part question – first asking about if they knew

about Zika, then asking if it made them worry or care, and third asking if they delayed the pregnancy because of it. The limitations of this question should be discussed.

RESPONSE:
This question is not a three-part question and was not asked in sequence to prior questions. This is an independent question that asks for decision making based on perceived risk hence reflects behavior rather than knowledge.

- Table 3-; Since recruitment was conducted in the clinics could their have been a change in the type of information provided given clinicians knew the study was ongoing?

RESPONSE:
We agree with the reviewer that there is a possibility of change in the behavior of clinicians over time. Clinicians neither directly involved in the study nor and knew about the specific items asked in the survey. Thus, we assume that they did not change their communication behavior. We have added this issue under limitations, see page 24.
