# Supplementary material for: Communicating the risk of contracting Zika virus to low income underserved pregnant Latinas: A clinic-based study
Source: PLoS One. 2020 Nov 20;15(11):e0241675. doi: 10.1371/journal.pone.0241675 (PMC7679023; doi:10.1371/journal.pone.0241675)
Supplement: Attachment — Submitted filename: Response to reviewers.docx [file pone.0241675.s002.docx]

PLOS one review 2 Sept 2020

PONE-D-19-24828R1
Communicating the Risk of Contracting Zika Virus to Low Income Underserved Pregnant Latinas: A Clinic-based Study

Responses to Reviewer's Questions (see yellow highlighted text)

Please use the space provided to explain your answers to the questions above. You may also include additional comments for the author, including concerns about dual publication, research ethics, or publication ethics. (Please upload your review as an attachment if it exceeds 20,000 characters)

Reviewer #2:

Page 4
line 48	Replace “and” with “who” Done see line 55
line 49
State “Latin America” rather than “South America” since some foreign born participants were from Central America done Line 56

Page 6
line 96
Reword to depict that there is a paucity of data on pregnant Latinas, state why they are an important population to study. Underserved pregnant Latinas are at higher risk for…(references) see lines 112-114

Page 7
Lines 118-129
Authors do not provide inclusion and exclusion criteria for the study. For example, if literacy level was low, were the participants excluded from completing the survey? If participants as Brazilian or Spanish, were they excluded due to Spanish-language and/or European origin? We have now specified inclusion criteria lines 152-158

Page 8
lines 136-139
Please provide more details as to how potential participants were identified as “Latino.” Did they self-identify? Did the staff identify them based on their medical records? Did the staff ask them to self-identify for the purposes of this study? More detail is needed regarding how the ethnicity of the participants was determined for study inclusion. Provide a statement explaining the training of clinic staff to obtain informed consent. We have now specified inclusion criteria lines 152-158

Lines 151-159
Please provide more detail on how study participants completed surveys and how many questions the survey included. Were these completed with pencil and paper, link to online survey, etc. And if participants had a low literacy level, did they have a partner/friend/relative help them complete it or was it administered by trained study personnel in an interview format? More information is also needed on the survey responses and measures. Please describe how awareness, knowledge, travel behavior and information sources were measured in the survey. For example, were any of the questions open-ended, ranking, Likert scale items, etc. Details are now included UNDER MEASURES lines 182-217

Page 11
Lines 207-209
“Significantly higher proportion of 208 single mothers (57.8%) reported untimed/unwanted pregnancy compared to mothers who live with 209 their partner/spouse (38.9%) (2 = 8.58, df = 1, p=.003).”
This statement should be removed from the paper. It does not contribute to the author’s research questions, nor does it provide data for rich discussion. Further, it perpetuates racial/ethnic stereotypes. This sentence is now removed.

Page 12
Lines 241-242
Remove qualifier “surprisingly.” The authors did not collect data on where geographically the conception occurred, therefore it cannot be assumed that they felt ZIKV was a risk that contributed to family planning. removed

Page 16
Table 2
”§Adjusted for education and income” – Education and Income often indicate the same measure (multicollinearity), health care access. Research question states to examine only “education” among the demographics listed. If multicollinearity did not exist, either add income into research question. Although, the authors have a stronger variable to that also measures health care access, clinic type. This variable is more pertinent in this analysis to establish differences (if any) between patients at both clinics.
We tested for association between income and education before and they were not associated (p=.07). So we had included both of them in the model. However, we also ran logistic models for all dependent variables without income and the main results remained the same. So, we will go with the current Table 2 as is in the paper. As suggested by the reviewer, we have now included income in the research question.

Page 17
Lines 304-306
“Results indicate that the community health center (FQHC) neither actively inquired about mothers’ travel plans (2 =12.88; df=2; p = .002) nor warned about travelling to places where Zika virus was active (2 = 5.45; df=1; p = .02).”
In this statement, authors report that providers did not actively inquire about travel plans, however, please clarify that the question the participants were asked stated “Physician/nurse specifically asked about travel history or travel plans to a place where Zika virus outbreak is active.” Therefore, participants responded to the question of “travel to a place where Zika virus outbreak is active,” not just travel plans. Caution with interpretation of responses from this question. First, assumes that the patient knows where Zika virus is active when women in the younger age group possessed less knowledge on Zika in general. Second, the question is specific about “travel history or travel plans to a place where Zika virus outbreak is active,” not just travel plans in general. We have clarified this statement by adding appropriate terms. 374-376

Page 19
Lines 324-328 & Figure 2
It is unclear how the sources of information were assessed. Was this a select all that apply type of question or was it a ranking? We have clarified this under the measures section, see lines 204-208.

Lines 334-344
Same concern with this section. How were these questions measured or were they open ended? If so, is it possible that the participants had survey fatigue and skipped this section? We have clarified this under the measures section, see lines 209-217.

Page 20
Lines 353-355
“Although there was an awareness of the negative impact on the babies, some women did not worry or care to delay pregnancy, in particular among mothers who planned their pregnancy.”

Authors do not provide data supporting this statement in either the tables or figures, except for one statement regarding “Fifty-five percent of mothers planned for current pregnancy”. I suggest it be removed. We have now deleted this sentence.

Line 364

Suggesting the HCPs “must” address misinformation is not reasonable. Health care providers each have roles, but they can collaborate with local health departments if misinformation is spreading through communities. Health departments are equipped with epidemiologists and health educators to address such problems, and HCPs have an opportunity to stimulate change. We have now added a sentence to reflect this idea. 434-438.

Lines 364-366
“Factual knowledge must be imparted through prenatal clinics, and particularly those serving low-income vulnerable populations.”

This statement contradicts the finding regarding sources of information regarding Zika. Study participants reported that their main sources of information include the Media/Internet (65%), physician nurse (32.3%), and clinic (20%). Therefore, even if clinics provided “factual knowledge” the populations being served will not be reached. This recommendation should be removed. If possible replace with recommendations based on public health campaign messaging literature. Replaced it with new literature lines 507-513.

Line 368-369
“Our study indicated that single mothers are at a higher risk of not having such critical knowledge.”

Data to support this statement is not presented. None of the p-values in table 2 are significant for the questions when examined by “couple status.” Now it is corrected Lines 440-442

Page 21
Lines 380-383
Authors argue that because over half of mothers reported an unintended pregnancy, they could have travelled to high risk “counties” (I think authors meant countries). This is a big leap given that they only obtained travel information from a limited number of mothers. I would remove text starting with “Nearly…” in line 380 ending in “…sex.” In line 383. We have now deleted and revised the sentence.459-463.

Lines 384-385
Authors argue that mothers did not care to postpone a pregnancy despite the Zika outbreak. However, the study was conducted in a community that was at low risk for Zika at the time. Therefore, this statement should be removed or the authors need to provide stronger argument that this community was at high risk. Underserved pregnant Latinas are not at high risk for Zika due to their ethnicity, rather, the community outbreak status determines what the level of risk is for pregnant women. We have deleted and revised the text. Lines 459-463

Page 22
Lines 400-402
Remove statement “These groups are viewed as hard-to-reach populations, not only because of language barrier and lower trust in media, but also because they report relatively low use of various media channels.”

This statement has no reference. See previous statements from line 95-97. “Hard to reach” is not a justifiable reason to study a community/population. Additionally, internet media reaches this community very effectively. The reviewer misunderstood the meaning of the term, and also provided a reference 486-487.

Lines 402-404
Please expand the implications suggested. Lines 490-494

Lines 411-415
Authors argue that HCPs need to understand the sources of information patients get information from, which this study identified as internet being the primary source for information on Zika among pregnant Latinas. However, authors then argue that accessibility is an issue, which contradicts study findings. Lastly, the “Internet should be considered a substitute” statement is quite strong, given that the internet can refer to a multitude of sources for information. Please clarify and consider providing a recommendation that HCPs can suggest to patients as a good source of information regarding Zika.
revised text in Lines 504-508

Page 23 Line 420
Remove “targeted” and insert “patient” changed line 513

Lines 423-424
Suggest how HCPs can accomplish offering culturally appropriate Zika risk counseling. Expand this recommendation. We have added relevant text in lines 518-525.

Lines 432-433
See previous statements from line 95-97 and 400-402. “Hard to reach” is not a justifiable reason to study a community/population. Additionally, internet media reaches this community very effectively. We have clarified the term hard-to-reach (see explanation in lines 486-487)

Line 433
Please provide a more definitive statement rather than stating the “sample is ‘expected’ to be inclusive of 85% of pregnant Latinas in the county.” We have now corrected lines 538-539.

Page 25
Lines 486-487
See previous statements from line 95-97, 400-402, and 486-487. “Hard to reach” is not a justifiable reason to study a community/population. Now changed to “underserved” line 596

Page 26
Lines 489-490
I would remove this statement. Deleted A quick search yielded several studies. Of note, see study by Winneg et. al., Risk Analysis, Vol. 38, No. 12, 2018.

Lines 492-496
I recommend removing implications for COVID-19 until there is enough scientific evidence to explain how COVID-19 puts pregnant women at risk. However, the implications for dissemination of information is still applicable. We have added a sentence to reflect that research on this topic is still evolving. Lines 602-603.

Page 27
Lines 512-513
HCPs consists of a variety of health professionals. Suggesting that HCPs need training and preparation is ok, but I suggest that the conclusion provide more explicitly who HCPs can collaborate with to make this feasible.

We added the term nursing staff to clarify who specifically should be trained lines 630-631.

Reviewer #3: I did not participate in the first review, however it appears that the authors fully addressed the first reviewers concerns. This is a god paper and a relevant topic. I would like to call out a few minor issues to address before being published:

1) Line 48, there is a small typo - "and" should be changed to "who" DONE
2) Line 50/ 51 - denoted traveled to "by" March 2016. Should this be "in"? Is this time period of a single year or several years. Can you clarify? Also, what is meant by "passengers"? Passengers sounds like they arrived by train, plane or car however I don't see anything indicating the mode of transportation. PROVIDED CLARIFICATION
3) I recommend being consistent throughout the paper with calling it ZIKV. There are many instances where it goes back and forth between the abbreviation and Zika. CHANGED WHERE IT IS APPROPRIATE
4) Lines 92-93 - where are the other two clinics located? It would be nice to know a little more about the other two clinics. Also, there is a small typo on line 93, the word "the" needs to be inserted before Texas-Mexico border. DONE
5) Line 120 - typo in Cameron County (not Cameroon) DONE
6) Line 140 - typo, should be "they" waited or "waiting" DONE
7) Line 167 - typo, should read "at" least 8 weeks DONE
8) Lines 258-259 contains a redundant statement DONE
9) Line 338 - typo, need to add the word "they" to became pregnant DONE
10) Line 382, typo, should be "countries", not counties DONE

This research is commendable in that it identifies important gaps in knowledge transfer in particularly vulnerable women. Comparing between FQHCs and private clinics is key as it illustrates how each have areas to be improved.
