# Supplementary material for: Communicating the risk of contracting Zika virus to low income underserved pregnant Latinas: A clinic-based study
Source: PLoS One. 2020 Nov 20;15(11):e0241675. doi: 10.1371/journal.pone.0241675 (PMC7679023; doi:10.1371/journal.pone.0241675)
Supplement: Attachment — Submitted filename: Response to reviewers.docx [file pone.0241675.s003.docx]

PLOS one review 3

PONE-D-19-24828R2
Communicating the Risk of Contracting Zika Virus to Low Income Underserved Pregnant Latinas: A Clinic-based Study
PLOS ONE

Thank you for the reviewers’ comments. We have made requested changes

Additional Editor Comments (if provided):
Please check the references once more. E.g. in lines 441-448, the references are not correctly ordered and sometimes you are using brackets and sometimes square brackets.

All the brackets have now been corrected to square brackets. The references were inserted using Mendeley and I am not sure why some a few of the brackets are different.

Reviewers' comments and response:
In view of the comment provided by Reviewer 2 (see below) we request to consider whether it is possible to describe why your study population is a hard-to-reach group - or whether this group is just underrepresented in previous research.

Reviewer #2: Thank you for the opportunity to review the author’s revisions on the study titled, “Communicating the risk of contracting Zika virus to low income underserved pregnant Latinas: A clinic-based study.” The article is very much improved. The topic is timely and relevant. It adds to the body of work with ethnically diverse populations in the U.S.

I have one major comment:

Lines 442-444, lines 489-490 We have now deleted this term. Changed it to underrepresented in research
Authors attempted to justify the term “hard-to-reach” using a citation that refers to mostly geographical challenges in accessing a community. This citation also describes sampling techniques.

 I realize that this term is a nuanced by fields. I leave it to the editor to decide if this term is appropriate for the journal - hence my minor revision recommendation.
